# Recent Advances in Biological and Technological Research of Fresh Fruit and Vegetable: 2nd Edition

**DOI:** 10.3390/foods14173029

**Published:** 2025-08-29

**Authors:** Zhongqi Fan

**Affiliations:** College of Food Science, Fujian Agriculture and Forestry University, Fuzhou 350002, China; fanzqi@fafu.edu.cn

Fresh fruits and vegetables are highly favored by consumers, owing to their abundance in various nutrients, such as vitamins, minerals, and dietary fiber [1,2]. However, as perishable commodities, they exhibit vigorous physiological metabolism postharvest, rendering them susceptible to quality deterioration during storage and transportation, which consequently results in substantial economic losses [3,4]. Thus, the application of preservation technologies is crucial for maintaining the quality of fruits and vegetables, extending their shelf life, and minimizing economic losses [5,6]. This Special Issue compiles 13 research articles and 1 review, which collectively focus on the preservation techniques, physiological characteristics, and biological mechanisms associated with fresh fruits and vegetables.

For the application of physical preservation technologies, cushioning packaging has been shown to effectively delay the quality deterioration of Chinese olive fruits during cold chain transportation and extend their storage life. The cushioning packaging enhanced the antioxidant capacity of the Chinese olive fruits, inhibited the activity of enzymes related to membrane damage, and promoted the accumulation of key metabolites in the phenylpropanoid pathway and flavonoid biosynthesis pathways. It thereby regulated the balance of reactive oxygen species (ROS), alleviated oxidative stress during cold chain transportation, and effectively maintained the cell membrane integrity of the fruits [contribution 1]. Additionally, as a physical preservation method with low energy consumption and no chemical residues, the high-voltage electrostatic field (HVEF) preservation technology delayed the senescence process of sweet cherry fruits by inhibiting their respiratory metabolism. HVEF also effectively inhibits the growth of pathogenic bacteria by regulating the structure and diversity of the fungal community on fruit surfaces, thereby enhancing the disease resistance of fruits and significantly improving the quality of sweet cherries during cold storage [contribution 2].

Chemical preservation technologies have been reported to extend the shelf life of fruits and vegetables by inhibiting microbial growth, delaying physiological metabolism, and preventing oxidative deterioration [7,8]. Within this Special Issue, studies have demonstrated that both individual and combined applications of calcium (Ca) and boron (B) significantly enhance the break force and elasticity of ‘Liuyuezao’ pummelo fruits, while markedly reducing the incidence of fruit cracking during the expansion period. The treatment of Ca and B down-regulated the cell wall-degrading enzymes’ gene expression and their corresponding enzymatic activities, thereby effectively delaying cell wall degradation and maintaining its structural integrity and stability. Furthermore, these treatments could increase the contents of sugar and vitamin C, reduce organic acid content, and consequently enhance the overall internal quality of the fruits [contribution 3].

1-methylcyclopropane (1-MCP), a highly effective ethylene inhibitor, has been extensively utilized for preserving fresh fruits and vegetables [9]. Research included in this Special Issue indicated that the 1-MCP pre-cutting treatment effectively suppressed the accumulation of soluble quinone content (SQC) by modulating the activities of peroxidase (POD) and polyphenol oxidase (PPO), as well as influencing the metabolism of phenolic substances, thereby significantly reducing flesh browning in fresh-cut nectarines during refrigeration. Furthermore, this treatment enhanced the antioxidant capacity of fruits, decreased membrane lipid peroxidation, preserves cell membrane integrity, and thus effectively delays the deterioration of fruit quality. In addition, it promoted the synthesis of aroma-related volatile compounds during storage, increased their total content, and further improved the overall sensory quality of fresh-cut nectarines [contribution 4]. Additionally, zinc oxide nanoparticles (ZnO NPs), as efficient antioxidant agents, could significantly enhance the antioxidant capacity of litchi fruits and inhibit the activities of browning-related enzymes, thereby delaying both peel browning and decay during storage time and maintaining fruit quality [contribution 5].

6-benzylaminopurine (6-BA), an artificially synthesized cytokinin, is commonly employed to delay aging in various horticultural crops [10,11]. Wang et al. found that 6-BA could regulate the activities of respiratory-related enzymes and the expression of related genes in the glycolysis pathway (EMP), tricarboxylic acid cycle (TCA), cytochrome pathway (CCP), and pentose phosphate pathway (PPP). Thus, 6-BA treatment inhibited the decline of respiratory rate, reduced energy and material consumption, and effectively delayed the senescence of cabbage leaves postharvest [contribution 6].

Acidic electrolyzed water (AEW) is a green fungicide generated through the electrolysis of low-concentration sodium chloride solution. It holds considerable potential for application in the postharvest preservation of fruits and vegetables [12,13]. The authors analyzed the impact of AEW with different available chlorine concentrations (ACCs) (20, 40, 60, and 80 mg L^−1^) on the disease occurrence of wampee fruits. Results indicated that treating wampee fruits with AEW (pH 2.5) with 40 mg L^−1^ ACC could improve the activities of disease resistance-related enzymes, promote the accumulation of lignin, effectively enhance the disease resistance of the fruits, inhibit the occurrence and development of diseases, and significantly improve their storage quality [contribution 7].

Melatonin (MT) is a natural compound that has been extensively utilized in the preservation of postharvest fruits and vegetables due to its disease-resistant, anti-aging, and prominent antioxidant properties [14]. Wan et al. investigated that melatonin could improve loquat fruits’ cold tolerance through the regulation of ROS generation and metabolic balance. The research found that loquat fruit treatment with 50 μmol L^−1^ MT could effectively enhance the antioxidant capacity, improve cellular efficiency in scavenging DPPH radical scavenging ability, and effectively suppress excessive ROS accumulation [contribution 8].

Moreover, studies have shown that combining MT treatment (200 μmol L^−1^, soaked for 15 min) with controlled atmosphere (CA, 2–3% O_2_ + 15–16% CO_2_) effectively enhanced the antioxidant capacity and delayed the senescence of lemons during cold storage. This synergistic approach offered valuable insights for the development of efficient, low-residue preservation technologies, demonstrating significant potential in maintaining fruit and vegetable quality and extending shelf life [contribution 9].

At the molecular level, Guo et al. systematically elucidated the impact of sulfur dioxide (SO_2_) fumigation on the quality of postharvest ‘Munage’ table grapes through integrating metabolomics and transcriptomics analyses. Their findings suggested that SO_2_ might activate specific transcription factors (TFs), thereby modulating the expression of genes involved in flavonoid metabolism. This regulatory network promoted the accumulation of protective flavonoid metabolites, which effectively suppressed postharvest decay and softening processes in ‘Munage’ table grapes, thus preserving fruit quality [contribution 10].

For biological methods, essential oils (EOs) are an important biological control resource extracted from aromatic plants, acting as broad-spectrum antibacterials and efficient antioxidants [15]. EOs could control postharvest diseases by directly inhibiting the growth of pathogenic bacteria and inducing the host to produce systemic resistance [16]. Mrvova et al. evaluated the antibacterial effects of 10 EOs (625 μL L^−1^) on the pathogen *Penicillium olsonii* of cherry tomatoes using the gas diffusion method. Their findings revealed that EOs derived from thyme (*Thymus vulgaris* L.), oregano (*Origanum vulgare* L.), wild thyme (*Thymus serpyllum* L.), marjoram (*Satureja hortensis* L.), and savory (*Satureja hortensis* L.) exhibited significant antifungal activity, effectively suppressing the development of P. olsonii on cherry tomatoes. This study offered an important theoretical basis for the development of EOs-based postharvest preservation technologies [contribution 11].

In recent years, biological control agents (BCA) have shown extensive application prospects in postharvest preservation of fruits and vegetables due to their environmentally friendly and highly safe characteristics [17]. Among various microbial antagonists, yeast strains exhibit lower nutritional requirements and superior tolerance to harsh environmental conditions. They are capable of inhibiting the growth of a wide range of pathogenic bacteria and demonstrate excellent compatibility with industrial processing techniques [18]. Li et al. successfully isolated three epiphytic yeasts (*Papiliotrema terrestris*, *Rhodosporidium glutinis*, and *Hanseniaspora uvarum*) with potential biocontrol properties from the healthy blueberry fruits and systematically analyzed the inhibitory effects of these yeast strains on *Alternaria alternata* and *Botrytis cinerea* in blueberry fruits, as well as their potential antagonistic mechanisms. The results indicated that three yeasts with different biological control mechanisms could significantly inhibit fungal diseases caused by *A. alternata* and *B. cinerea* on blueberry fruits. Moreover, these yeast strains improved the quality of blueberry fruits, providing new options for their utilization in preserving fruits and vegetables [contribution 12].

Another study has revealed the inhibitory effect of another microbial antagonist, nystatin, on the growth and development of *B. cinerea* on table grapes postharvest. Its main mechanism of action was by disrupting the permeability of the cell membrane of *B. cinerea*, causing the leakage of cellular contents and ultimately leading to cell death. In addition, nystatin could interfere with the expression of genes related to ribosomes and mitochondrial composition and biosynthesis, affecting the function of the ubiquitin-proteasome system (UPS) responsible for protein degradation, and inhibiting autophagy activity, thereby significantly weakening the pathogenic ability of *B. cinerea* [contribution 13].

Pitaya (*Hylocereus*) is native to tropical and subtropical regions. It has garnered considerable popularity in the global consumer market due to its distinctive appearance and substantial nutritional value. Although pitaya is a non-climacteric fruit, its metabolic activities remain relatively active after harvest, making it susceptible to microbial infection. During storage, it is prone to softening, water loss, shrinkage, and nutrient loss, which significantly reduces its commercial value and preservation period [19,20]. This review systematically examined the key issues in postharvest quality maintenance of pitaya, establishing a comprehensive research framework that spans from fundamental biological characteristics to practical preservation technologies. Firstly, the authors analyzed the differences in biological characteristics among various varieties of pitaya and elucidated the fundamental patterns of postharvest physiological and biochemical changes, thereby revealing the underlying mechanisms driving quality deterioration. Secondly, the authors elaborated on the pathogenic bacteria responsible for pitaya diseases and the characteristics of these diseases. In addressing core issues such as postharvest quality deterioration and pathogen infection, the authors conducted a systematic evaluation of the application efficacy of various preservation technologies, including physical, chemical, and biological approaches. Finally, the authors comprehensively analyzed the molecular mechanisms underlying pitaya ripening and senescence, and systematically summarized recent advances in resistance induction techniques. Collectively, this review offers robust theoretical foundations and practical technical guidance for improving postharvest quality of pitaya [contribution 14].

This editorial has summarized the research progress in fruits and vegetables preservation technologies reported in this Special Issue. According to the physiological characteristics of different fruits and vegetables and the primary mechanisms of postharvest deterioration, the researchers have developed a diversified preservation technology system. For physical methods, applications include cushioning packaging and HVEF. For chemical treatments, applications include Ca and B treatments, 1-MCP, 6-BA, AEW sterilization, SO_2_ fumigation, MT, and ZnO NPs. Notably, researchers have proposed a synergistic preservation strategy that integrates MT pretreatment with CA, offering novel insights into the development and application of postharvest preservation technologies for fruits and vegetables. Regarding biological methods, the focus is on the development and application of plant EOs and highly effective microbial antagonists. Taken together, this Special Issue enhances our understanding of physiological changes and molecular mechanisms in postharvest fruits and vegetables, and provides advanced technologies to extend their shelf life and maintain quality.

## References

[1] Du L., Huang X., Li Z., Qin Z., Zhang N., Zhai X., Shi J., Zhang J., Shen T., Zhang R. (2025). Application of Smart Packaging in Fruit and Vegetable Preservation: A Review. Foods.

[2] Jiang Q., Zhang M., Xu B. (2020). Application of ultrasonic technology in postharvested fruits and vegetables storage: A review. Ultrason. Sonochem..

[3] Dai O., Wang Y., Li M., Hong W., Lei X., Ye Z., Pang J., Sun Y. (2025). Post-harvest precooling technology and fresh-keeping mechanism of fruits and vegetables: A review. Postharvest Biol. Technol..

[4] Zuo X., Wang J., Li Y., Zhang J., Wu Z., Jin P., Cao S., Zheng Y. (2025). Recent advances in high relative humidity strategy for preservation of postharvest fruits and vegetables: A comprehensive review. Food Chem..

[5] Wang D., Zhang M., Li M., Lin J. (2024). Fruits and vegetables preservation based on AI technology: Research progress and application prospects. Comput. Electron. Agric..

[6] Dwibedi V., Kaur G., George N., Rana P., Ge Y., Sun T. (2024). Research progress in the preservation and packaging of fruits and vegetables: From traditional methods to innovative technologies. Food Packag. Shelf Life.

[7] Ma L., Zhang M., Bhandari B., Gao Z. (2017). Recent developments in novel shelf life extension technologies of fresh-cut fruits and vegetables. Trends Food Sci. Technol..

[8] Maringgal B., Hashim N., Tawakkal I., Mohamed M. (2020). Recent advance in edible coating and its effect on fresh/fresh-cut fruits quality. Trends Food Sci. Technol..

[9] Nguyen L., Zsom T., Dam M., Baranyai L., Hitka G. (2019). Evaluation of the 1-MCP microbubbles treatment for shelf-life extension for melons. Postharvest Biol. Technol..

[10] Liu J., Meng F., Jiang A., Hou X., Liu Q., Fan H., Chen M. (2024). Exogenous 6-BA enhances salt tolerance of Limonium bicolor by increasing the number of salt glands. Plant Cell Rep..

[11] Zhang L., Shi X., Hou H., Lin Q., Zhu S., Wang G. (2023). 6-Benzyladenine Treatment Maintains Storage Quality of Chinese Flowering Cabbage by Inhibiting Chlorophyll Degradation and Enhancing Antioxidant Capacity. Plants.

[12] Wang F., Lin Y., Xu Y., Ba Y., Zhang Z., Zhao L., Lam W., Guan F., Zhao Y., Xu C. (2023). Mechanisms of acidic electrolyzed water killing bacteria. Food Control.

[13] Zhang W., Cao J., Jiang W. (2021). Application of electrolyzed water in postharvest fruits and vegetables storage: A review. Trends Food Sci. Technol..

[14] Feng B., Kang D., Sun J., Leng P., Liu L., Wang L., Ma C., Liu Y. (2022). Research on melatonin in fruits and vegetables and the mechanism of exogenous melatonin on postharvest preservation. Food Biosci..

[15] Elshafie H. (2022). Plant Essential Oil with Biological Activity. Plants.

[16] Angane M., Swift S., Huang K., Butts C., Quek S. (2022). Essential Oils and Their Major Components: An Updated Review on Antimicrobial Activities, Mechanism of Action and Their Potential Application in the Food Industry. Foods.

[17] Sharma R., Singh D., Singh R. (2009). Biological control of postharvest diseases of fruits and vegetables by microbial antagonists: A review. Biol. Control.

[18] Ma Y., Wu M., Qin X., Dong Q., Li Z. (2023). Antimicrobial function of yeast against pathogenic and spoilage microorganisms via either antagonism or encapsulation: A review. Food Microbiol..

[19] Liu R., Gao H., Chen H., Fang X., Wu W. (2019). Synergistic effect of 1-methylcyclopropene and carvacrol on preservation of red pitaya (*Hylocereus polyrhizus*). Food Chem..

[20] Fan P., Huber D., Su Z., Hu M., Gao Z., Li M., Shi X., Zhang Z. (2018). Effect of postharvest spray of apple polyphenols on the quality of fresh-cut red pitaya fruit during shelf life. Food Chem..

